# Endoscopic large-balloon dilation alone versus endoscopic sphincterotomy plus large-balloon dilation for the treatment of large bile duct stones

**DOI:** 10.1186/1471-230X-13-15

**Published:** 2013-01-17

**Authors:** Jae Chul Hwang, Jin Hong Kim, Sun Gyo Lim, Soon Sun Kim, Sung Jae Shin, Kee Myung Lee, Byung Moo Yoo

**Affiliations:** 1 Department of Gastroenterology, Ajou University School of Medicine, San-5, Woncheon-dong, Yongtong-gu 443-721 Suwon, Korea

**Keywords:** Common bile duct stones, Endoscopic sphincterotomy, Large-balloon dilation

## Abstract

**Background:**

Endoscopic sphincterotomy (EST) combined with large-balloon dilation (LBD) has been proposed as an alternative to manage large bile duct stones. However, recent reports indicate that LBD without EST may be safe and effective in this setting.

**Methods:**

One hundred thirty-one patients with large common bile duct (CBD) stones 12 mm in size or larger underwent LBD alone (*n* = 62) or EST plus LBD (*n* = 69) for lithotripsy. The therapeutic outcome and complications were reviewed and compared.

**Results:**

There were no differences between the two groups with regard to age, size and number of stones, or bile duct diameter. The LBD alone group (mean age, 70.4 years) and the EST plus LBD group (mean age, 68.2 years) had similar outcomes in terms of overall successful stone removal (96.8% vs. 95.7%, *P* = 0.738) and complete stone removal without the need for mechanical lithotripsy (80.6% vs. 73.9%, *P* = 0.360). Complications in the LBD alone and EST plus LBD groups were as follows: pancreatitis (6.5% vs. 4.3%, *P* = 0.593), impaction of basket and stone (0% vs. 1.4%, *P* = 0.341), and perforation (0% vs. 1.4%, *P* = 0.341).

**Conclusions:**

LBD alone may be a simple, safe, and effective alternative to EST plus LBD in relatively aged patients with large CBD stones, and it can simplify the procedure compared with EST plus LBD.

## Background

Endoscopic retrograde cholangiopancreatography (ERCP) has become one of the most important techniques in the treatment of bile duct stones. It is usually combined with endoscopic sphincterotomy (EST) to extract bile duct stones using a standard balloon or basket catheter. Alternatively, endoscopic papillary balloon dilatation (EPBD) has been proposed for this indication because it is thought to preserve the function of the sphincter of Oddi and lessen the complications seen with EST, such as hemorrhage and perforation [1-4]. EPBD is technically easier than EST, especially if sphincterotome control is difficult, the margin for cutting is limited, or the appropriate cutting direction is in question [5]. However, EPBD has been associated with a higher risk of pancreatitis after ERCP [6-8].

Large bile duct stones appear to be more difficult to remove with conventional methods, such as EST and EPBD. Therefore, extraction of large bile duct stones may require mechanical lithotripsy (ML) as an adjunctive procedure, which likely lengthens the procedure time. A number of studies have been conducted using large-balloon dilation (LBD) after adequate EST to extract large bile duct stones [9-13]. In those studies, the authors suggested that EST plus LBD might lower the risk of postprocedure pancreatitis by directing balloon dilation toward the bile duct rather than the pancreatic duct [9-13]. However, recent studies have shown that LBD without preceding EST is safe and effective in patients with large common bile duct (CBD) stones [14,15]. We conducted the present study to compare the therapeutic outcome and complications between LBD alone and EST plus LBD for the treatment of large bile duct stones.

## Methods

The ERCP database at our institution was searched for prospectively collected data on patients with large bile duct stones who underwent LBD from March 2004 to April 2009. During the study period, 2665 ERCPs were performed at our institution. The patients were identified from the database using a search query and the medical records of the patients were reviewed using a standardized data entry form. From March 2004 to February 2008, LBD was routinely performed with EST, while LBD alone (without EST) was performed from March 2008 to April 2009. LBD without EST was introduced into this hospital in March 2008 and used for the treatment of large bile duct stones. We have conducted a prospective, randomized, comparative study to validate LBD without EST as an effective and safe treatment for endoscopic removal of large bile duct stones since May 2009. We analyzed the data before and after the omission of EST to investigate its effect on the success of stone clearance and complications. Patients with visualized bile duct stones ≥12 mm in maximum transverse diameter were included. Exclusion criteria were (1) bleeding diathesis, (2) prior EST or EPBD, (3) Billroth II or Roux-en-Y anatomy, (4) distal extrahepatic bile duct stenosis, (5) acute pancreatitis, and (6) intrahepatic bile duct stones. Based on these criteria, 62 patients were included in the LBD alone group and 69 patients were included in the EST plus LBD group. This study was approved by our institutional review board, and informed consent was obtained from all patients for the endoscopic procedures performed.

**Figure 1:**
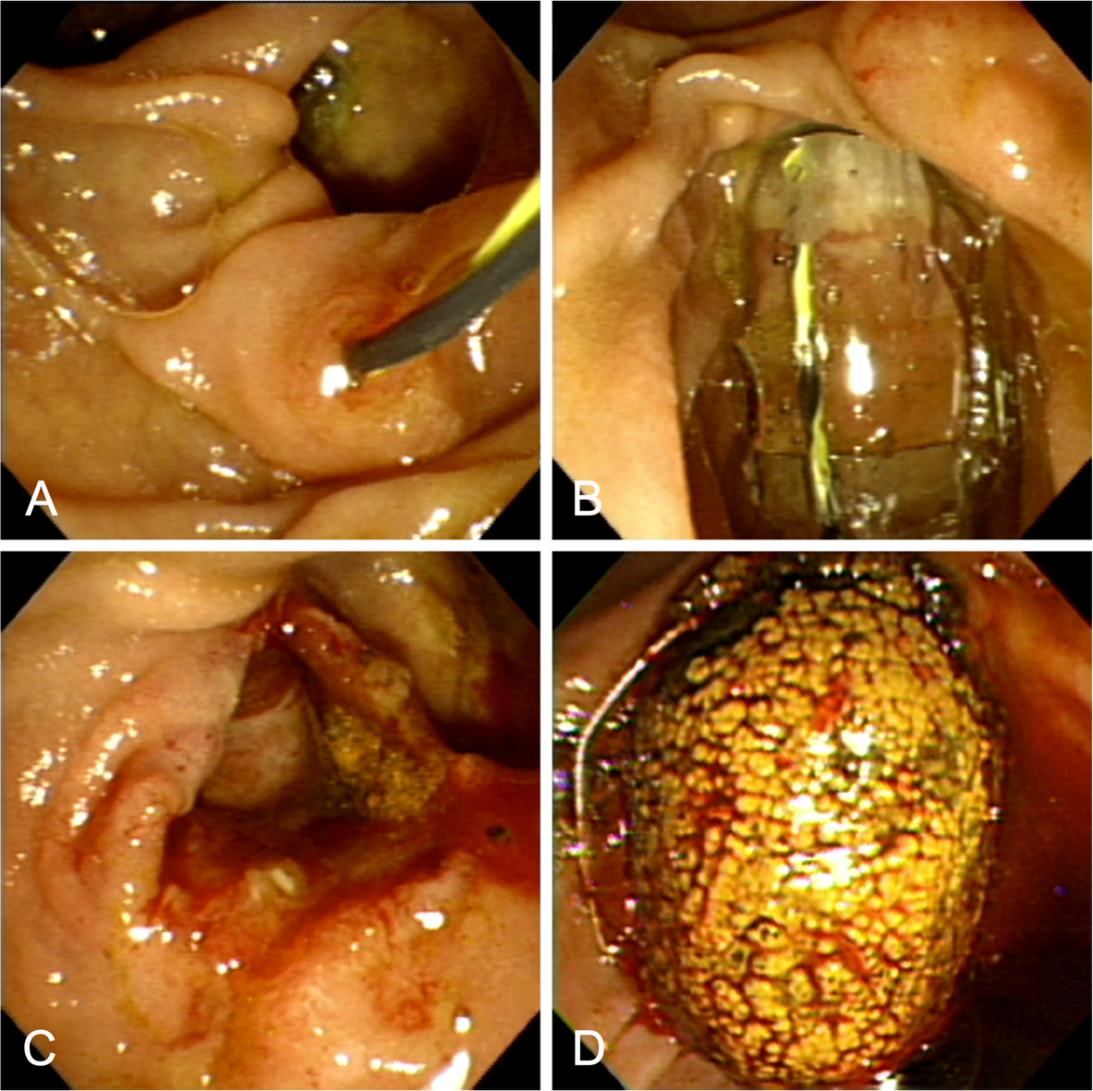
**Endoscopic view of large-balloon dilation without biliary sphincterotomy. A**. Guidewire positioned across the papilla. **B**. Large balloon inflated across the papilla without preceding endoscopic sphincterotomy. **C**. Markedly dilated papilla after large-balloon dilation. **D**. Large stone extracted with a basket through the dilated papilla.

**Figure 2:**
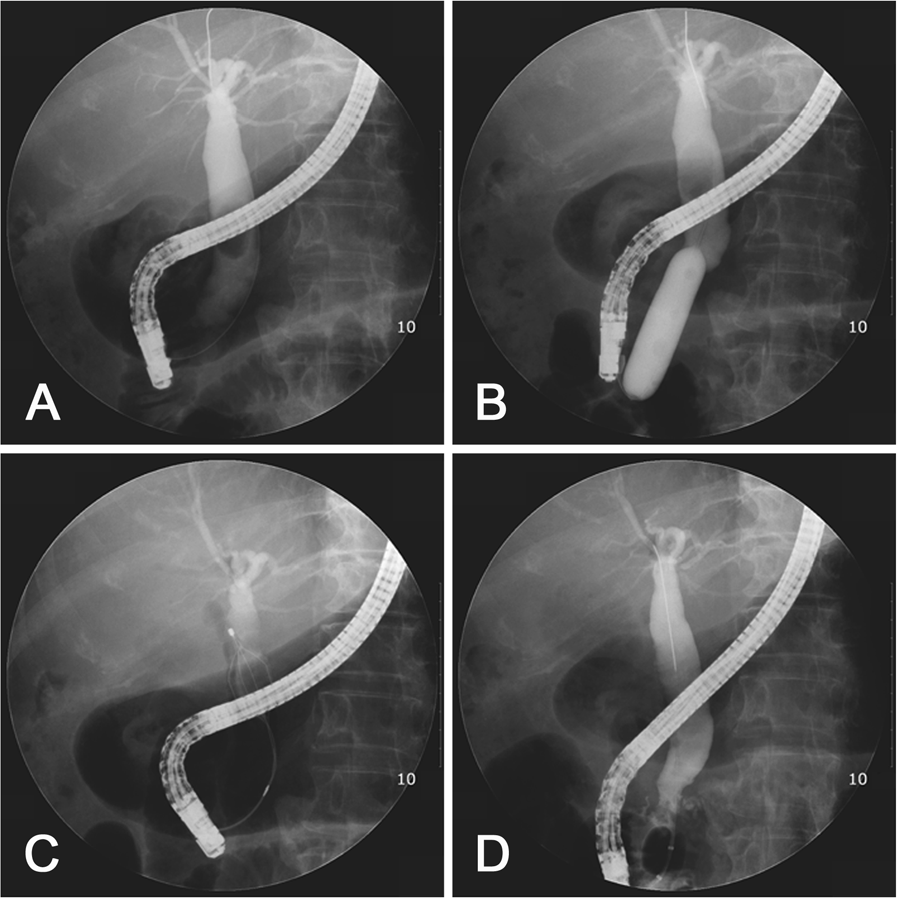
**Fluoroscopic view of large-balloon dilatation without biliary sphincterotomy. A**. Cholangiogram demonstrating a large stone within the dilated bile duct. **B**. Large balloon inflated across over guidewire. The diameter of the balloon was selected according to the diameter of the stone and of the bile duct proximal to the tapered segment under fluoroscopic guidance. **C**. The stone was captured in a basket. **D**. Cholangiogram after complete stone removal showed no residual filling defect in the bile duct.

ERCP was performed with side-viewing endoscopes (Olympus JF-240 or TJF-240; Olympus Optical Co., Ltd., Tokyo, Japan). Each patient was sedated with a standard dose of midazolam, propofol, and meperidine. After the CBD was selectively cannulated using a sphincterotome, an initial cholangiogram was taken. Diameters of the bile duct and stones were measured during ERCP and corrected for magnification using the external diameter of the duodenoscope’s distal end as a reference. In the EST plus LBD group, EST was performed before LBD from the orifice of the papilla proximally to the transverse fold (minor EST). Wire-guided hydrostatic balloon catheters (Boston Scientific Microvasive, Cork, Ireland) that can be dilated to the three distinct diameters listed on the package and hub labels were positioned across the major papilla with the balloon mid-portions placed at the biliary sphincter. The balloon was then gradually inflated to the pressure corresponding to the smallest balloon diameter with dilute contrast medium until the waist of the balloon had disappeared under fluoroscopic guidance. Thereafter, the pressure for inflation of the balloon was gradually increased until the desired dilation was achieved. Once the dilation to the desired diameter was achieved, the balloon was maintained in position for 60 seconds and then deflated and removed. The balloon diameters used were 12 to 20 mm, and the diameter of the balloon was selected according to the sizes of the stones and bile duct proximal to the tapered segment under fluoroscopic guidance. The bile duct stones were removed with a Dormia basket or retrieval balloon (Figures 1 and 2). A mechanical lithotripter was used to fragment the stones when standard methods failed to remove the stones, even after LBD.

Technical success was defined as complete removal of all CBD stones after LBD without the need for ML. Serum amylase, total bilirubin, and alkaline phosphatase levels; complete blood counts; and abdominal radiographs were checked before procedures and on the following day to monitor for complications such as bleeding, perforation, acute pancreatitis, and acute cholangitis. Complications were evaluated according to 1991 consensus guidelines [16] defining post-ERCP pancreatitis as persistent epigastric pain of >24 hours with a ≥3-fold elevation in serum amylase concentration after the procedure.

Statistical analysis was performed using the chi-square test or Fisher’s exact test for categorical parameters and Student’s *t* test for continuous variables. Analyses were performed using SPSS 12.0 (SPSS Inc., Chicago, IL), with quantitative data presented as mean ± standard deviation. Statistical significance was set at a *P* value of <0.05.

## Results

Demographic data for the 131 patients included in the study are summarized in Table 1. There were no significant differences between the two groups with regard to age, periampullary diverticulum, size and number of stones, or bile duct diameter.

**Table 1:** Baseline clinical characteristics of the patients

	**LBD alone**	**EST plus LBD**	***P *value**
	**(*n*** **= 62)**	**(*n*** **= 69)**	
Gender (male/female)	23/39	33/36	0.215
Age (years)	70.4 ± 10.9	68.2 ± 10.5	0.902
Periampullary	33 (53.2)	38 (55.1)	0.832
diverticulum, no. (%)			
Cholecystectomy, no. (%)	16 (25.8)	18 (26.1)	0.971
Bile duct stones			
Size (mm)^a^	15.7 ± 3.3	16.5 ± 4.2	0.182
Number	2.3 ± 1.6	2.8 ± 1.8	0.109
Bile duct size (mm)^b^	20.5 ± 4.4	21.4 ± 4.6	0.996

LBD: Large-balloon dilation; EST: Endoscopic sphincterotomy.

Continuous variables are expressed as mean ± standard deviation.

^a ^Maximum transverse diameter of the largest stone.

^b ^Maximum transverse diameter of the bile duct proximal to the tapered segment of the distal common bile duct.

LBD with or without EST was successfully performed in all patients. The mean diameter of the balloon used for LBD was 15.9 mm (range, 12–20 mm) for the LBD alone group and 16.2 mm (range, 12–20 mm) for the EST plus LBD group (*P* = 0.444). The overall stone clearance was ultimately similar between the LBD alone group (96.8%) and the EST plus LBD group (95.7%) whether or not ML was used (*P* = 0.738) (Table 2). The technical success rate was 80.6% in the LBD alone group and in 73.9% in the EST plus LBD group (*P* = 0.360). Complete stone clearance was not feasible during the first session in nine patients (five patients in the LBD alone group vs. four patients in the EST plus LBD group, *P* = 0.608). Failure of complete duct clearance occurred in five patients (two patients in the LBD alone group, three patients in the EST plus LBD group) despite the fact that ML was attempted. The causes of failure were stone impaction and incomplete stone capture with the basket. These patients underwent percutaneous transhepatic cholangioscopy to remove the stones, with the exception of one patient who required surgery in the EST plus LBD group.

**Table 2:** Results of bile duct clearance after large-balloon dilation

	**LBD alone (*n*** **= 62)**	**EST plus LBD (*n*** **= 69)**	***P *value**
Diameter of inflated balloon (mm)	15.9 ± 2.3 (12-20)	16.2 ± 2.5 (12-20)	0.444
Complete stone removal irrespective of whether ML was used, no. (%)	60 (96.8)	66 (95.7)	0.738
Complete stone removal without ML, no. (%)	50 (80.6)	51 (73.9)	0.360

LBD: Large-balloon dilation; EST: Endoscopic sphincterotomy; ML: Mechanical lithotripsy.

Continuous variables are expressed as mean ± standard deviation (range).

When the two study groups were further divided by success or failure of complete stone removal after LBD (without the need for ML), significant differences were observed with regard to the stone size (LBD alone: 14.6 ± 2.1 mm vs. 20.2 ± 3.5 mm, *P* < 0.001; EST plus LBD: 15.5 ± 2.9 mm vs. 19.4 ± 5.9 mm, *P* < 0.001) and the balloon/stone diameter ratio (LBD alone: 1.01 ± 0.10 vs. 0.82 ± 0.08, *P* < 0.001; EST plus LBD: 1.15 ± 0.19 vs. 0.90 ± 0.18, *P* < 0.001) (Table 3).

**Table 3:** Comparison of clinical characteristics between the success and failure groups

	**LBD alone (*n*** **= 62)**			**EST plus LBD (*n*** **= 69)**		
	**Success group (*n*** **= 50)**^**a**^	**Failure group (*n*** **= 12)**^**b**^	***P *value**	**Success group (*n*** **= 51)**^**a**^	**Failure group (*n*** **= 18)**^**b**^	***P *value**
Bile duct stones						
Size (mm)	14.6 ± 2.1	20.2 ± 3.5	<0.001	15.5 ± 2.9	19.4 ± 5.9	<0.001
Number	2.2 ± 1.7	2.9 ± 1.2	0.166	2.8 ± 1.8	3.0 ± 1.9	0.609
Balloon/stone diameter ratio	1.01 ± 0.10	0.82 ± 0.08	<0.001	1.15 ± 0.19	0.90 ± 0.18	<0.001

LBD: Large-balloon dilation; EST: Endoscopic sphincterotomy.

^a ^Complete stone clearance without mechanical lithotripsy.

^b ^Application of mechanical lithotripsy or failure to extract stone even after LBD.

Continuous variables are expressed as mean ± standard deviation.

Post-ERCP complications are summarized in Table 4. Rates of pancreatitis did not differ significantly between the two groups (6.5% vs. 4.3%, *P* = 0.593), and these cases were mild and self-limiting. Perforation was observed in one patient in the EST plus LBD group. This complication was found shortly after complete stone clearance, and the patient recovered uneventfully following surgical intervention. In another patient in the EST plus LBD group, the basket was broken on a hard stone during ML, and a portion of the broken basket was retained in the bile duct. The patient subsequently underwent bile duct exploration with a satisfactory outcome. No clinically significant hemorrhage occurred in either group.

**Table 4:** Complications in LBD alone and EST plus LBD groups

**Complications**	**LBD alone (*n*** **= 62)**	**EST plus LBD (*n*** **= 69)**	***P *value**
Pancreatitis	4	3	0.593
Bleeding	0	0	
Perforation	0	1	0.341
Embedded broken basket after ML	0	1	0.341
Total	4	5	0.858

LBD: Large-balloon dilation; EST: Endoscopic sphincterotomy.

## Discussion

In the current study, LBD without EST was as effective and safe as EST plus LBD in patients with large bile duct stones. To our knowledge, this is the first study to compare the efficacy and safety of LBD alone with EST plus LBD for the treatment of large bile duct stones.

EPBD was originally devised to extract CBD stones while minimizing damage to the sphincter of Oddi. However, the drawback of EPBD compared with EST is the more limited size of the papillary opening. Approximately 10% of bile duct stones are difficult to remove using conventional techniques, and for these patients, ML is generally the next step [17-19]. However, ML is time-consuming, has a potential for injury of the EST site or bile duct, and may be complicated by impaction of the stone-capturing basket. Moreover, because small stone fragments after ML may act as nidi for stone recurrence, ML is one of the risk factors for recurrent bile duct stones after endoscopic stone extraction [20]. The main purpose of LBD is to avoid or lessen the use of ML for removal of large CBD stones and to reduce complications that may be related to ML.

Ersoz et al. [9] first reported the use of EST followed by EPBD with large-diameter (12–20 mm) balloons as an alternative technique for bile duct stones that are difficult to remove by standard methods. Complete stone retrieval without ML was successful in 54 (93.1%) of 58 patients, and stone clearance was achieved by ML in 4 (6.9%) patients. Complications occurred in nine patients (15.5%), including two (3.4%) with mild pancreatitis. Several studies have since been conducted using EST plus LBD for large, difficult bile duct stones [10-13,21,22]. Figures for overall stone clearance have ranged from 95% to 100%, with ML required for 1% to 27%. Complication rates have also varied from 0% to 8.3%, with pancreatitis between 0% and 4.5%. In most previous studies using EST plus LBD for removal of large CBD stones [9-13,21,22], the authors suggested that this technique may be associated with a lower risk of pancreatitis because EST prior to LBD may result in separation between the pancreatic and biliary orifices, and it can guide the direction of balloon dilation toward the bile duct rather than the pancreatic duct during LBD. However, recent two studies indicate that LBD without EST may be safe and effective in patients with large CBD stones [14,15]. In a retrospective preliminary study [14], overall successful stone removal was achieved in 37 (97.4%) of 38 patients, and ML was required in 8 (21.1%) patients. A mild degree of postprocedure pancreatitis developed in only one (2.6%) patient. The authors proposed that a prior EST before LBD may not play an important role in the guidance of balloon dilation toward the bile duct. They also suggested that ML may induce papillary edema or spasms that may obstruct the pancreatic duct orifice. Thus, LBD may lower the incidence of pancreatitis by reducing the need for ML when removing large bile duct stones. In addition, because LBD is not performed on a nondilated CBD, which is one of the risk factors for post-ERCP pancreatitis, LBD may not carry the same risk of postprocedure pancreatitis as EPBD with a balloon catheter diameter of ≤10 mm for the removal of CBD stones [14]. In another retrospective study [15], overall complete stone clearance was achieved in 229 (92.7%) of 247 patients, and ML was needed in 39 (15.8%) patients while retrieving the stones. There were nine (3.6%) complications, including two (0.8%) cases of mild pancreatitis. In the present study, the rates of overall stone clearance and complete stone removal without ML were similar between the two groups (96.8% vs. 95.7%, *P* = 0.738; and 80.6% vs. 73.9%, *P* = 0.360, respectively). The pancreatitis rates were similar between the two groups (6.5% vs. 4.3%, *P* = 0.593), and all cases were mild and self-limiting. The progressive decline in pancreatic exocrine function with aging may protect older patients from pancreatic injury, and one meta-analysis comparing EST and EPBD for bile duct stones demonstrated that age of <60 years was one of the factors related to a higher rate of pancreatitis in patients with EPBD [23,24]. Therefore, the relatively old age of the patients in the current study may explain these results.

An additional purpose of LBD is to reduce complications by avoiding full-incision EST (major EST) in patients with large CBD stones. Although the reported bleeding rates from previous studies involving LBD range from 0% to 9% [9-15,21], several reports on the performance of major EST before LBD showed a relatively high incidence of bleeding (8.3%–9%) [9,11]. In this study, minor EST was performed before LBD in the EST plus LBD group and clinically significant hemorrhage was not noted in either group.

Other complications occurred in two patients in the EST plus LBD group. Perforation resulting from a duodenal wall tear opposite the major papilla occurred in one patient. It occurred during stone removal with a basket after LBD and ML and was caused by the tip of the duodenoscope. This complication was found shortly after complete stone removal. A basket impaction occurred in the other patient. The basket capturing the stone was broken during ML, and a portion of the broken basket remained in the bile duct. These complications were not related to LBD, and both patients recovered with surgical intervention.

Previous definitions of technical success have varied by publication [9-13,21]. To define technical success, the frequency of required examinations may be used, but is often subject to the endoscopist. Moreover, the goal of LBD in managing large CBD stones is to avert ML and its potential complications. In the present study, we defined technical success as complete removal of CBD stones by performing LBD without an additional procedure such as ML, and we did not take into account the number of endoscopic sessions. In a retrospective study of LBD alone for retrieval of large CBD stones [14], patients in the treatment failure group showed a tendency to have a greater transverse stone diameter and smaller balloon/stone diameter ratio than patients in the treatment success group (20.8 ± 6.5 mm vs. 16.7 ± 3.9 mm *P* = 0.077] and 0.80 ± 0.23 vs. 0.96 ± 0.19 *P* = 0.066], respectively). In another retrospective multicenter study of EST plus LBD for bile duct stone removal [13], the median maximum stone size in patients undergoing ML was significantly larger than that in patients who did not undergo ML (16.7 vs. 13.3 mm, *P* < 0.01). In this study, treatment failure was associated with larger transverse stone diameters compared with treatment success and smaller balloon/stone diameter ratios. These results suggest that ML is more frequently used with larger stone sizes and that using a balloon catheter with a diameter smaller than the maximum transverse diameter of the stone causes resistance at the ampullary opening during stone removal with a basket or retrieval balloon catheter. Thus, the diameter of the balloon should exceed the maximum transverse diameter of the stone, but not the diameter of the bile duct.

Our patient group is small and the study was limited by its retrospective nature. Moreover, our study included many older patients who may be related to a lower rate of postprocedure pancreatitis. Therefore, the efficacy and safety of LBD alone in relatively young patients with large CBD stones remains uncertain. Randomized studies comparing LBD alone and EST plus LBD should be conducted in order to confirm our results.

## Conclusions

LBD alone may be a simple, safe, and effective alternative to EST plus LBD in relatively aged patients with large CBD stones, and it can simplify the procedure compared with EST plus LBD.

## Competing interests

The authors declare that they have no competing interests.

## Authors’ contributions

JCH, JHK, and BMY participated in the conception and design of the study and performed the ERCP procedures; JCH, JHK, and SGL participated in the analysis and interpretation of the data; JCH, JHK, SSK, SJS, KML and BMY were responsible for pre- and post-procedure patient care; JCH and JHK were responsible for writing the manuscript and revising it critically for important intellectual content. All authors read and approved the final manuscript.

## Pre-publication history

The pre-publication history for this paper can be accessed here:

http://www.biomedcentral.com/1471-230X/13/15/prepub
